# 3D electron diffraction studies of synthetic rhabdophane (DyPO_4_·*n*H_2_O)

**DOI:** 10.1107/S2053229624007885

**Published:** 2024-09-04

**Authors:** Ercin C. Duran, Mohamed Ruwaid Rafiuddin, Yazhou Shen, Simon A. Hunt, Anamul Haq Mir, Alexander S. Eggeman

**Affiliations:** a Department of Materials, University of Manchester, M13 9PL, United Kingdom; b Department of Metallurgical and Materials Engineering, Istanbul Technical University, Türkiye; c School of Computing and Engineering, University of Huddersfield, HD1 3DH, United Kingdom; Rigaku Americas Corporation, USA

**Keywords:** crystal structure, 3DED, electron diffraction, rhabdophane, nanocrystal, superstructure, dysprosium phosphate

## Abstract

In this study, we report the results of continuous rotation electron diffraction studies of single DyPO_4_·*n*H_2_O (rhabdophane) nanocrystals. The diffraction patterns can be fit to a trigonal lattice (*P*3_1_21) with lattice parameters *a* = 7.019 (5) and *c* = 6.417 (5) Å. However, there is also a set of diffuse background scattering features present that are associated with a disordered superstructure that is double these lattice parameters and fits with an arrangement of water mol­ecules present in the structure pore. Pair distribution function (PDF) maps based on the diffuse background allowed the extent of the water correlation to be estimated, with 2–3 nm correlation along the *c* axis and ∼5 nm along the *a*/*b* axis.

## Abstract 2

3D ED studies of synthetic rhabdophane allow single-crystal structure solution from individual nanocrystals and provides insight into the disordered water arrangment in the material. This highlights the usefulness of this technique to study order/disorder at nanoscale and its potential in exploring the effects of irradiation-induced structural and phase changes at such scales in the near future.

## Introduction

Some of the naturally occurring monazite minerals (*e.g.* Monazite-Ce, CePO_4_) contain significant amounts of radioactive Th (>20 wt% ThO_2_) and U (up to 10 wt% UO_2_), and yet, these minerals are never found in the metamict (*i.e.* amorphous) state despite being exposed to self-irradiation damage (Nasdala *et al.*, 2020 ▸). Due to their excellent radiation damage tolerance and chemical durability, monazite (*RE*PO_4_, with *RE* = La–Gd; space group *P*2_1_/*n*) based waste forms are proposed as potential candidates for managing long-lived radionuclides (Terra *et al.*, 2003 ▸; Dacheux *et al.*, 2013 ▸). Several studies have been carried out in the literature investigating the effects of radiation damage and corrosion on the structure of synthetic monazites and it was shown that in aggressive corrosive media, a secondary phase called rhabdophane (*RE*PO_4_·*n*H_2_O, with *RE* = La–Gd) precipitates on the surface of the monazite ceramic (Arinicheva *et al.*, 2018 ▸; Zhao *et al.*, 2020 ▸). Rhabdophane has a low solubility constant and, as a result, possess good actinide retention properties which can help limit the release of actinides (including Th and U) into the biosphere (Du Fou de Kerdaniel *et al.*, 2007 ▸). Therefore, understanding its structure and properties is important for assessing the long-term performance of actinide-rich monazite waste forms.

The crystal structural determination of rhabdophane poses an inter­esting scientific problem and in the literature, efforts have been made to solve the crystal structure *via* powder X-ray diffraction (PXRD, laboratory- and synchrotron-based) and, more recently, *via* three-dimensional electron diffraction (3DED) tomography (Mayence *et al.*, 2014 ▸). It is important to note that the structure of rhabdophane has never been solved using single-crystal XRD due to the practical difficulties of growing a larger diffraction-quality crystal. In the late 1940s, using PXRD, Mooney (1948 ▸, 1950 ▸) first reported the crystal structure of rhabdophane (*RE*PO_4_·*n*H_2_O, with *RE* = Ce, La, Pr and Nd) and concluded that it (CePO_4_·*n*H_2_O) crystallizes in the hexa­gonal structure with open channels running along the hexa­gonal axis. In that study, Mooney assigned two possible space groups (*P*3_1_21 or *P*6_2_22) to account for the systematic absence (*i.e.* it was reported in that study that the 00*l* planes were absent unless *l* = 3*n*) in the diffraction pattern of CePO_4_·*n*H_2_O. Mooney suggested that the water mol­ecules could occupy the open channels in the structure; however, no attempt was made in that study to identify the location of the water mol­ecules.

The precise arrangement of water in the hydrated structure was not determined until high-resolution synchrotron-based X-ray diffraction (HRXRD) and thermogravimetric analysis (TGA) by Mesbah *et al.* (2014 ▸). This work proposed a significant reduction in the sym­metry of the structure to monoclinic (*C*2) with a structure analogous to partially hydrated calcium sulfate (this will be referred to as the monoclinic I structure). The relaxation of the structure was needed to accommodate several weak scattering features that could not be fit to the higher sym­metry of the Mooney structure. Importantly, the study validated the hydration level of RePO_4_·0.667H_2_O as the stable room-tem­per­a­ture com­position and proposed a configuration of the water mol­ecules in the structure pore. Further studies of dehydration of the material (Mesbah *et al.*, 2017 ▸) have also revealed changes in the material to a partially dehydrated monoclinic structure, RePO_4_·0.5H_2_0 (monoclinic II), and finally a fully dehydrated structure that fits the original trigonal form proposed by Mooney. There are excellent overviews of the structural and chemical variations of the monazite–rhabdophane–xenotime–churchite system (Rafiuddin *et al.*, 2022*a* ▸) that will not be repeated here, instead this report will focus on the hydrated rhabdophane structure only.

There has recently been a growth in the use of electron diffraction for structural studies of this kind, particularly where the high spatial resolution of electron microscopes allows individual nanocrystals to be studied within a powder sample (Kolb *et al.*, 2007 ▸, 2011 ▸), removing the need for large single-crystal samples and so providing an opportunity to study materials where growth of large crystals is either difficult or not possible. The expansion of this methodology in recent years (Gemmi *et al.*, 2019 ▸; Gruene *et al.*, 2018 ▸) is also of value for studying systems exhibiting com­plex structural variations, since the coexistence of phases or the presence of microstructural features (Krysiak *et al.*, 2018 ▸; Hua *et al.*, 2021 ▸) can introduce artefacts into ensemble approaches, such as powder analysis. Electron diffraction can, therefore, provide useful com­plimentary evidence for such materials. Moreover, it is especially useful in determining the position of water and even hy­dro­gen in the lattice (because of the strong Coulombic inter­action), something that can be very difficult with X-rays. The focus of this study is to revisit the structure of hydrated rhabdophane through the study of hydrated dysprosium phosphate.

## Experimental methods

The synthesis and structural characterization of the materials used in this work is covered in our earlier article (Rafiuddin *et al.*, 2022*b* ▸), but the synthesis protocol is nevertheless briefly described here. Rhabdophane-type DyPO_4_·H_2_O was synthesized *via* the precipitation method. The following precursors were used to synthesize DyPO_4_·H_2_O: Dy_2_O_3_ (Alfa Aesar Chemicals, 99.9%), H_3_PO_4_ (Alfa Aesar Chemicals, 85%), HNO_3_ (Acros Organics, 70%) and NaOH (Fischer Chemicals, analytical reagent grade, pearls). Prior to the synthesis, 100 ml of a 0.05 *M* solution of Dy(NO_3_)_3_ were prepared by dissolving 0.94 g of Dy_2_O_3_ in 10 ml concentrated nitric acid solution, followed by dilution of the clear solution to a 100 ml volume in a volumetric flask using deionized water. 1 *M* H_3_PO_4_ and 8 *M* NaOH solutions were also prepared prior to the synthesis. 30 ml of 0.05 *M* Dy(NO_3_)_3_ were added to a vessel containing 6 ml of 1 *M* H_3_PO_4_ solution under continuous stirring and the pH of the solution was raised to ∼1.5 using 8 *M* NaOH and was left stirring for 1 h. After 1 h, the pH was increased to 1.5 and then heated to 45 °C in an oven for 1 d (without any further stirring) and the resulting fine precipitates were wash­ed with deionized water and dried in air at 40 °C for 2 d.

A small fraction of the precipitate was dispersed in ethanol to obtain isolated single-crystal nanorods and then dispersed on to a standard TEM (transmission electron microscopy) carbon grid by pipetting a small drop of the liquid on to the grid.

Electron diffraction experiments were performed on a ThermoFisher Talos 200X TEM operated at 200 kV. Parallel beam illumination was used and a 10 µm C2 aperture inserted to minimize the beam size to approximately 800 nm diameter. The rotation range was limited by the overlap of the copper TEM grid at higher tilts and the usable range of +45 to −55° was achieved. The goniometer was rotated at approximately 1.12° s^−1^ and the diffraction patterns from individual crystals were recorded on a Quantum Detectors Merlin Quad hybrid pixel detector using continuous acquisition at 12-bit depth and 100 ms frame exposure.

Data was reconstructed and sectioned using the *PETS* software package (Palatinus *et al.*, 2019 ▸). The structure solution was performed using charge-flipping (implemented as *SUPERFLIP*) in *JANA* (Petricek *et al.*, 2014 ▸, 2023 ▸). Further diffraction pattern analysis was also performed using the Nanomegas ‘Automated crystal orientation mapping’ (*ACOM*) software (Rauch *et al.*, 2010 ▸) and through Python routines developed within the electron microscopy group at the University of Manchester. PDF analysis was performed using the ‘numpy’ Python library for array manipulation and Fourier transforms.

## Results

The rhabdophane sample com­prised rod-like crystals with a typical width of 50–100 nm and lengths in the range 800–1500 nm. This allowed a single rhabdophane nanocrystal of width 80 nm to be identified and isolated in the electron beam. The nanocrystal was favourably oriented with its long axis lying approximately along the tilt axis of the goniometer. This meant that the effective sample thickness did not vary significantly during the tilt experiment (though there was increased thickness of the supporting carbon film at high tilts). Alignment of the sample height and the sample position to the tilt axis was performed to minimize sample movement; however, the crystal moved partially out of the illumination for the final 1.6° of the tilt range. Despite this, a very high level of com­pleteness was achieved with 91% of the unique reflections with *d*-spacing greater than 0.7 Å was possible.

**Figure 1:**
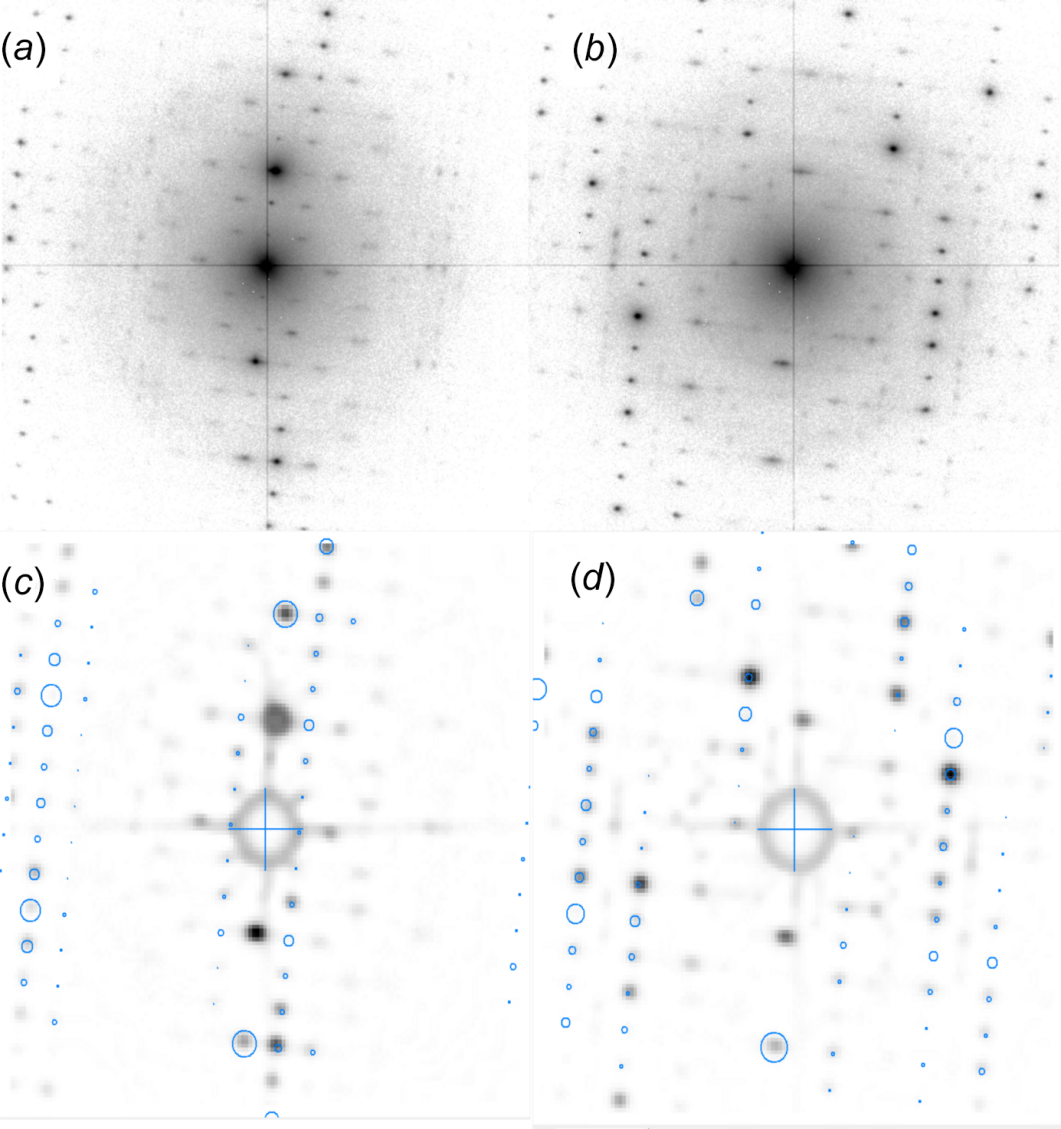
Example electron diffraction patterns [reproduced with log(Intensity)] from the rhabdophane sample. Parts (*a*) and (*b*) show patterns recorded close to the <100> and <110> crystal orientations, respectively [note the halo arising from the amorphous carbon support films is more pronounced because of the log(Intensity) display]. Parts (*c*) and (*d*) show *ACOM* orientation matches to the <740> and <860> template libraries, respectively.

Figs. 1 ▸(*a*) and 1(*b*) show representative patterns extracted from a continuous diffraction acquisition modified to show *log(Intensity)* to enhance structured diffuse scattering present in the patterns. These patterns lie close to the <740> and <860> crystal directions of the trigonal structure model of rhabdophane, respectively, determined by template matching using the automated crystal orientation mapping (*ACOM*) software (Rauch *et al.*, 2010 ▸). The patterns matching *ACOM* outputs are shown in Figs. 1 ▸(*c*) and 1(*d*), with blue circles overlaid to show the positions of the reflections in the kinematical template pattern. These orientations were chosen to lie relatively close (∼6° mis-orientation) to the [100] and [110] zone-axis orientations for the trigonal description of the rhabdophane crystal to provide clear matches to the simulated patterns but to be tilted far enough from the zone-axis orientation to show the reciprocal space between the zeroth and first Laue zones. What is notable is that beyond the clearly defined Bragg peaks indicated in Figs. 1 ▸(*c*) and 1(*d*), there is a clear structured diffuse background in the patterns (for com­parison, examples of the natural intensity patterns are shown in Fig. S1 in the supporting information).

The natural intensity patterns were then processed using the *PETS* software (Palatinus *et al.*, 2019 ▸) to analyse reciprocal space and reconstruct the reciprocal lattice. Automatic peak finding was performed at an intensity threshold (*I*/σ) of 5. The relative strength of the Bragg reflections (and the relative weakness of the structured background) is highlighted by the fact that, of the recovered reflections, 92% (1283/1381) fit a trigonal cell with the lattice parameters given in Table 1 ▸ that agrees well with the rhabdophane structure from Mooney (1948 ▸).

This indexing only fits the stronger reflections found in the experimental data. The weak background could not be isolated using a conventional peak-finding method and attempts to do so introduced artefacts from the background scattering around the direct beam, as well as from the amorphous carbon support film visible in the patterns.

To extract the weak background features from the diffraction data, reciprocal space sections equivalent to 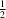
*kl* and *hk*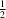 of the original trigonal cell were extracted (these can be thought of as the 1*kl* and *hk*1 sections of a 2*a* × 2*b* × 2*c* supercell). The effective increase in dimensions of the cell implied by this goes some way towards the increased size of the monoclinic superstructures proposed by Mesbah *et al.* (2014 ▸, 2017 ▸), though without any reduction in the sym­metry of the cell. Other fractional reciprocal space sections were also tested to see if there was any additional higher-order diffuse scattering present, but no structured scattering was found beyond that found in the two sections mentioned previously.

**Figure 2:**
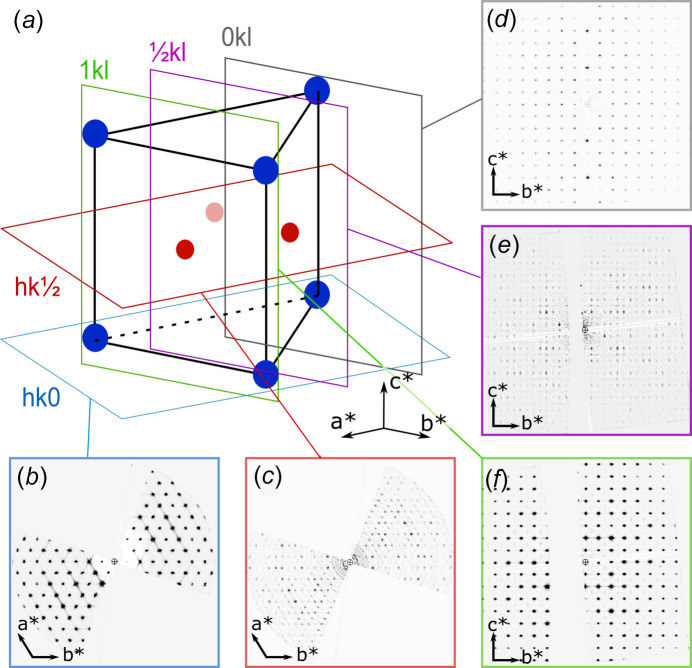
Reciprocal space sections from rhabdophane electron diffraction data. (*a*) A schematic of a partial unit of the reciprocal lattice with the trigonal reciprocal lattice indicated by blue circles and the disordered features marked by red circles. Individual reciprocal space sections corresponding to (*b*) *hk*0, (*c*) *hk*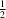, (*d*) 0*kl*, (*e*) 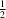
*kl* and (*f*) 1*kl* are indicated by planes in part (*a*).

These sections, along with those containing the 0*kl*, 1*kl* and *hk*0 scattering vectors, are shown in Fig. 2 ▸. It should be noted that, since the tilt axis was aligned almost along the crystal *c* axis, the ‘missing wedge’ (arising from the finite tilt range in the TEM) is highly evident in the *hk*0 and *hk*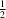 reciprocal space sections, with some portion of this manifesting as the empty bands in the 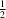
*kl* and 1*kl* sections. Fig. 2 ▸(*b*) shows the expected hexa­gonal arrangement of reflections; however, there are some weak diffuse streaks visible in the background of the section, suggesting at least one other additional type of disorder in the structure. These reinforce the threefold sym­metry of pattern, even though much of the streaking is absent because of the missing wedge region of the slice. The ‘*hk*
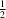
’ section in Fig. 2 ▸(*c*) shows a set of scattering features lying at the midpoint between the Bragg peak positions in the ‘*hk*0’ section. Importantly, there are three of these arranged as a triangle in each reciprocal space subunit and so these also maintain the threefold rotation sym­metry along this axis, rather than supporting a reduction to a lower sym­metry.

The ‘0*kl*’ section in Fig. 2 ▸(*d*) shows the expected rectangular arrangement of Bragg peaks. The 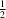
*kl* section in Fig. 2 ▸(*e*) confirms the position of the additional background peaks having basis vectors half the length of both *a* and *c* (but with alternating systematic rows along *c*), hence the general *hkl* indices are of the type 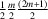 (where *m* is an integer). For the 1*kl* section, the diffuse streaking seen in the *hk*0 section is evident and the additional scattering features are located at the centres of the rectangles of Bragg reflections, meaning the background scattering has *hkl* indices of type 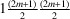, which matches that expected from Fig. 2 ▸(*c*). The same centring is seen in the 0*kl* section, but the visibility of the background features is extremely low, making it almost impossible to visualize in this figure.

These are summarized in the schematic reciprocal lattice representation in Fig. 2 ▸(*a*). Here the trigonal substructure is indicated by the blue positions at the corners of the cell, while the background features are indicated by the red positions at the prismatic face centres. The position of each reciprocal space section is shown by the coloured planes. The suggestion here is that the doubling of all three unit-cell parameters within the trigonal space group is the most accurate description of the material being studied, though the superstructure is not fully correlated, as will be described in the next section.

Inspection of the reciprocal space sections showing background features, suggests that the additional scattering is not as sharp as that seen in the 0*kl* section, attributed only to Bragg scattering. Instead, these are considerably elongated along the c* direction, suggesting that they are arising from or affected by a breakdown of long-range order in the structure.

**Figure 3:**
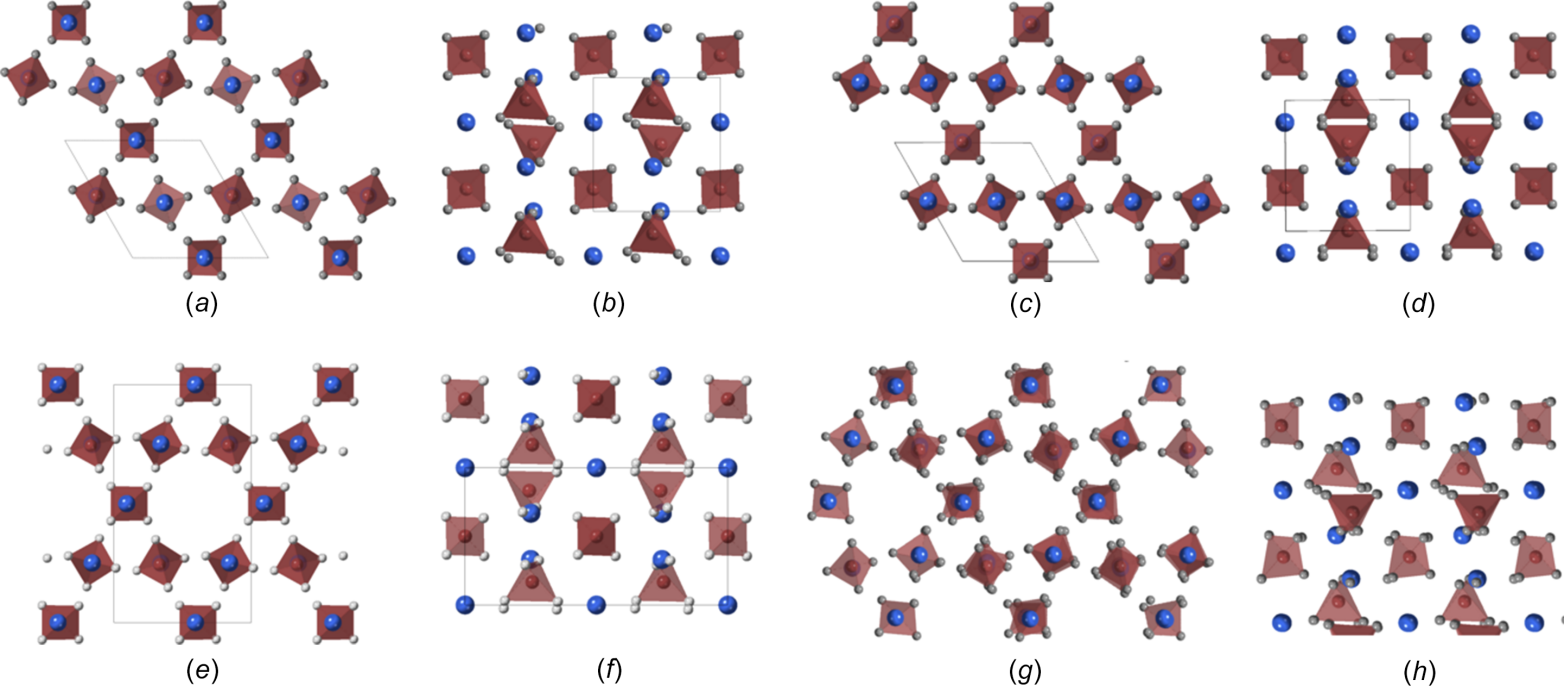
Structure projections of rhabdophane. Parts (*a*) and (*b*) are the solutions for the experimental data viewed along [001] and [100], respectively. Parts (*c*) and (*d*) are the same projections for the trigonal structure, parts (*e*) and (*f*) for the monoclinic II structure, and parts (*g*) and (*h*) for the monoclinic I structure (note the monoclinic I cell is larger than the others, so only a part of it is included for com­parison). Blue refers to the Dy ion, grey to the O ion and brown to the PO_4_ tetra­hedron.

Finally, structure solution of the tilt-series data (only including the strong Bragg scattering and not the weak background) was performed in *JANA* (Petricek *et al.*, 2014 ▸, 2023 ▸) using the ‘*SUPERFLIP*’ charge-flipping algorithm. The solution was made in the trigonal space group *P*3_1_21, with the unit-cell parameters given in Table 1 ▸. The [001] and [100] projections of the solved structure are shown in Figs. 3 ▸(*a*) and 3(*a*), respectively, with the Dy ion and PO_4_ tetra­hedron positions indicated. For com­parison, the equivalent projections of the trigonal (Mooney, 1948 ▸), monoclinic II (Mesbah *et al.*, 2017 ▸) and monoclinic I (Mesbah *et al.*, 2014 ▸) structures are included in Figs. 3 ▸(*c*)–3(*h*), respectively.

## Discussion

The solved structure [Figs. 3 ▸(*c*) and 3(*b*)] incorporates the sym­metry of the original trigonal structure from Mooney (1948 ▸, 1950 ▸), but exhibits similar displacements of the cations from the monoclinic structures of Mesbah *et al.* (2014 ▸). Importantly, the structure, when viewed along the trigonal [100] direction [Fig. 3 ▸(*b*)], shows the clear zigzag of the cation positions (resulting from rotations of the PO_4_ tetra­hedra) that was determined for the monoclinic I structure [Fig. 3 ▸(*h*)]. The distortion of the hexa­gonal channel at the centre of Fig. 3 ▸(*a*) is therefore similar to that seen in the monoclinic I structure [Fig. 3 ▸(*g*)], tending towards a triangular pore, rather than the hexa­gonal pores seen in the trigonal and monoclinic II [Figs. 3 ▸(*c*) and 3(*e*), respectively]. It is worth reiterating that the displacements of the cations shown in Figs. 3 ▸(*a*) and 3(*b*) occur within the sym­metry of the trigonal structure, they do not require the reduction of sym­metry of the crystal. It should be noted that the solution did not return positions for the oxygen or hy­dro­gen in the water mol­ecules.

Given the difference in the pre­sent­ed structures, the monoclinic II [Figs. 3 ▸(*e*)–3(*f*)] structure variant will no longer be considered for com­parison. The evidence against the monoclinic transformation is strengthened if simulated electron diffraction patterns for this structure are considered. In the case of the reduction in sym­metry, there would be a large number of (relatively weak) reflections introduced into the major zone axes.

**Figure 4:**
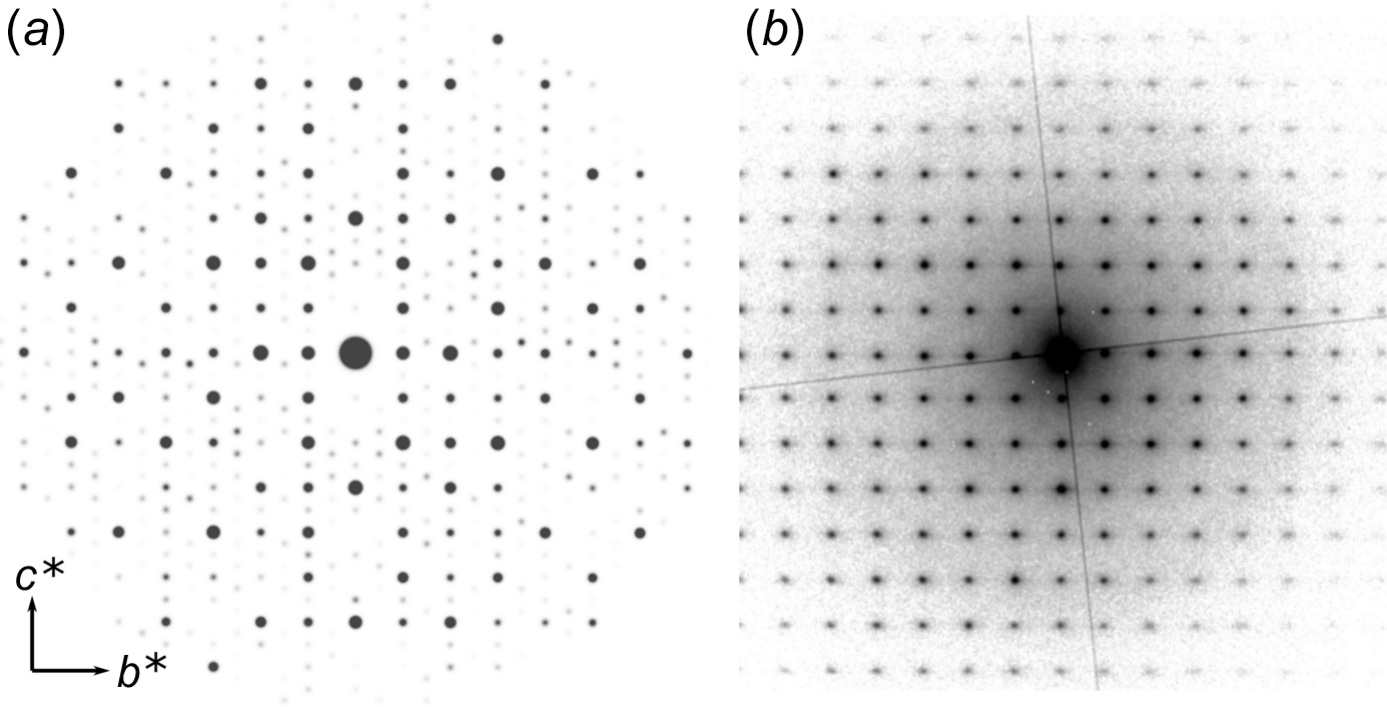
Electron diffraction patterns (*a*) simulated from the monoclinic crystal structure and (*b*) experimental data [log(Intensity)].

The kinematical simulated [100] diffraction pattern is shown in Fig. 4 ▸(*a*), outlining the expected location of any additional scattering attributed to the monoclinic distortions. The highest gain example of the equivalent zone-axis diffraction from the experimental acquisition is shown in Fig. 4 ▸(*b*). The experimental pattern is clearly not kinematical as there is evidence of dynamical scattering (such as the relatively strong 001 and 002 reflections); however, there is no trace of the superstructure reflections that would be expected from the monoclinic structure. The only additional scattering is the faint presence of the 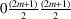-type reflections (particularly 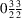 and 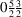).

The diffuse features in the experimental data help to develop the description of the material. In Mesbah *et al.* (2014 ▸), the move to monoclinic sym­metry is partly driven by weak reflections that could not be fit to the rhombic sym­metry proposed by Mooney. However, these weak peaks can be indexed using the background scattering seen in the experimental data (particularly Figs. 2 ▸(*c*) and 2(*e*). Specifically, scattering to 2θ angles of 15.4 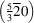, 16.2 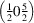, 16.3 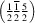, 16.5 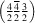 and 16.8° 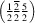 are all consistent with the experimental data pre­sent­ed. Furthermore, that study also reported changes in the lineshapes of the Bragg reflections, that can be explained by the superposition of both the Bragg and the diffuse scattering features seen in the electron diffraction data that have nearly identical scattering angles.

**Figure 5:**
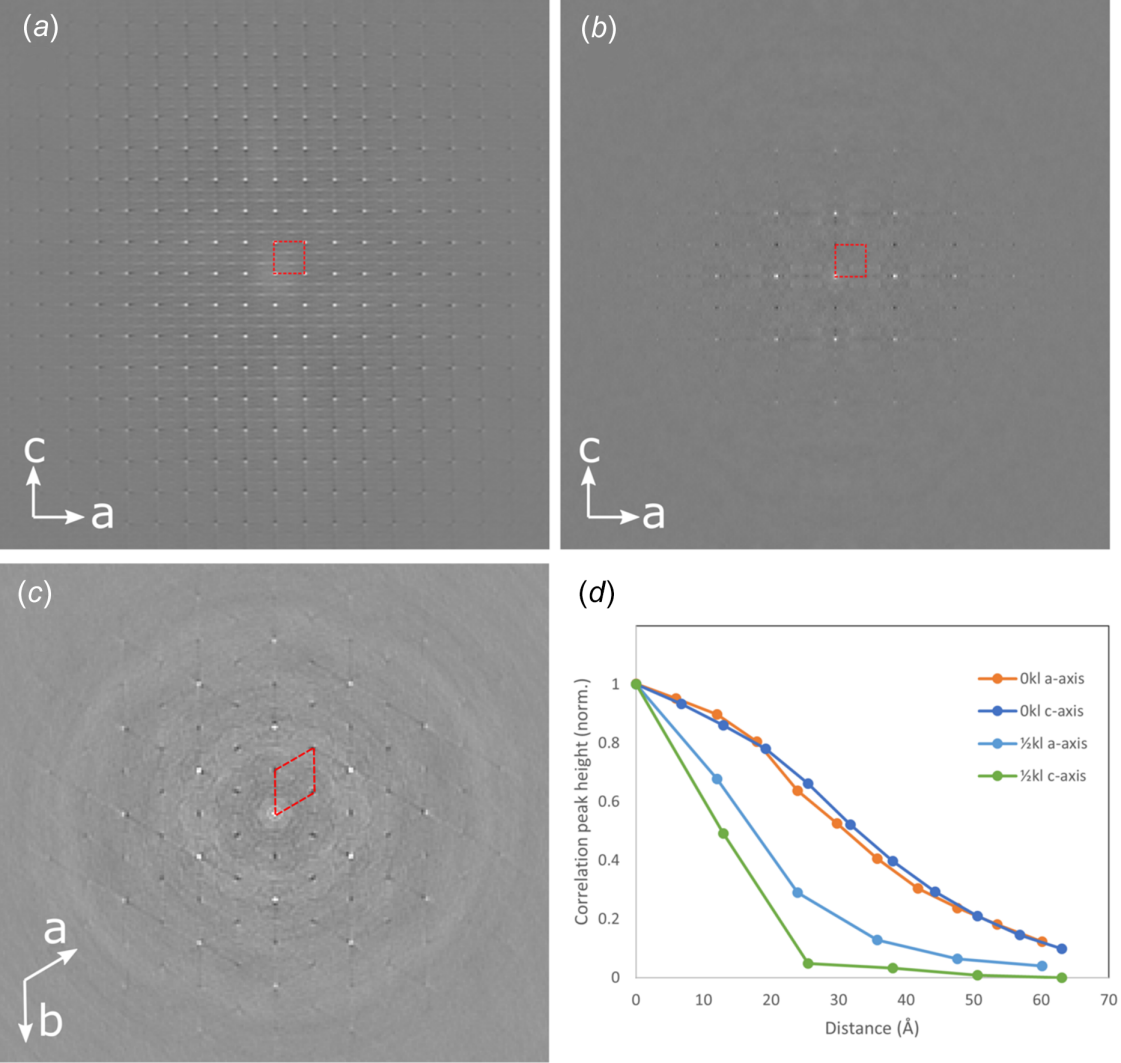
PDF analysis of reciprocal space sections corresponding to (*a*) 0*kl*, (*b*) 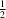
*kl* and (*c*) *hk*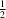. Part (*d*) shows the normalized correlation peak intensity along the *a* and *c* directions in the 0*kl* and 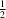
*kl* maps. Overlaid in red is the projection of the rhabdophane unit cell in all cases.

To study the diffuse character of the background scattering in more detail, a pair distribution function (PDF) analysis of the reciprocal space sections was performed, and the results shown in Fig. 5 ▸. Fig. 5 ▸(*a*) shows a PDF map produced from the 0*kl* reciprocal space section. This is almost entirely com­prised of Bragg reflections and so strong positive correlation peaks are seen to extend across the map. The correlation of each atom with its equivalent site in adjacent cells gives rise to the approximately square arrangement of strong peaks in this map, corresponding to the unit-cell projection (this is indicated in all of the PDF maps by a red dotted line). The fact that each sublattice (both Dy and P) has cations with *c* coordinates separated by approximately 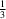 explains the strong bands of correlated vectors at *c*/3 and 2*c*/3 through the cell. Given the much higher atomic number of the cations, it is almost impossible to determine correlations involving the O atoms in the structure.

By contrast, the PDF of the 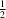
*kl* appears quite different and since this map excludes any long-range order (Bragg reflections) it can be inter­preted as a Δ-PDF map (Weber & Simon­ov, 2012 ▸). In these maps, the white contrast indicates en­hancements in the correlation (increased numbers of inter­atomic vectors above the average), while the dark con­trast is decreased correlation (or fewer inter­atomic vectors). In general, there is little or no correlation related to the heavy ion (rare-earth or phospho­rus) positions within the projected cell; instead all of the correlation peaks are at multiples of the projected cell dimensions. Along both *a* and *c*, there is a negative correlation peak at the first projected cell length and a strong enhancement of the vectors at twice the projected cell length (12.2 and 12.97 Å).

The positive peak at 2*c* suggests that there is a general ordering of atoms in alternating unit cells along the *c* axis. A similar behaviour is seen along *a*, particularly when the PDF of the *hk*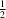 section [Fig. 5 ▸(*c*)] is considered. Here the vectors corresponding to the first neighbour cells (along all vectors equivalent to *a*) are negatively correlated, but there is a strong positive correlation in all of the second neighbour cells.

These observations fit a model where the background arises from the water mol­ecules in the pore running through the structure. The monoclinic I structure describes the water arrangement in the hydrated structure. Here the water mol­ecules within the central pore were reported to form hy­dro­gen bonds with two of the six DyO_8_–PO_4_ polyhedra chains bounding the pore, creating a zigzag arrangement of water mol­ecules that repeats over four unit cells of the fundamental trigonal structure. From the results reported here, we suggest that a similar zigzag arrangement of water mol­ecules appears to repeat only every two cells (hence the background features describe the negative correlation at a distance of *c* and a positive correlation at a distance of 2*c*). The possibility of this type of diffuse scattering arising from other atomic displacements seems unlikely. The DyO_8_ and PO_4_ coordination polyhedra are all edge sharing, making a simple rigid unit displacements or rotations less likely, or subject to the same sym­metry operations as the overall crystal. An independent distortion of one polyhedron would likely alter many of the heavy ion coordinates and so, in turn, would likely affect multiple heavy ion-ion vectors that lie within the projected cell [as seen in Fig. 5 ▸(*a*)], leading to additional changes in correlations within the individual unit cells, and these simply do not appear in the diffuse PDF maps [Figs. 5 ▸(*b*) and 5(*c*)].

The negative correlation along *a*, seen in both Figs. 5 ▸(*b*) and 5(*c*), suggests that the water mol­ecules in adjacent pores form hy­dro­gen bonds with a different subset of DyO_8_–PO_4_ polyhedra chains to those in the first pore, and only in the second neighbour shell does the water bonding revert to the original configuration (possible configurations of ordered water fitting hexa­gonal/trigonal sym­metry and anti-phase boundaries between ordered domains are pre­sent­ed in Fig. S2 of the supporting information). This fits with the crystal exhibiting hexa­gonal/trigonal sym­metry rather than the monoclinic sym­metry where all of the water chains bond to the equivalent DyO_8_–PO_4_ polyhedra chain in each pore. The structure is also in good agreement with the water arrangement seen in the partially hydrated calcium sulfate structure (Schmidt *et al.*, 2012 ▸) used to develop the original monoclinic model.

The disordered character of this behaviour is seen in more detail in Fig. 5 ▸(*d*), which shows linetraces taken along the *a* and *c* directions in Figs. 5 ▸(*a*) and 5(*b*). For the Bragg peaks (0*kl* section), the PDF shows strong correlation (that extends over many tens of Ångström), but which gradually decreases at higher distances as thermal displacements and finite beam-convergence effects lead to a breakdown in correlations. By contrast, the diffuse scattering in the 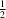
*kl* section data shows a far more rapid breakdown in the periodic order in the water substructure. It appears that, on average, the ordering of water in the *c* axis is highly disordered with the correlation falling to almost zero after only two of the water ‘zigzag’ arrangements (2–3 nm). Given there are three equivalent sets of DyO_8_–PO_4_ polyhedra chains that the water can hy­dro­gen bond to, and two possible arrangements within each set of chains, there are a large number of possible ways to achieve the water arrangement in the structure. Domains of the order of a few nanometres that can randomly fit these different arrangements would explain the disorder reported in this study.

Along *a*, the ordering of hy­dro­gen bonding in the second neighbour pores extends to around twice this distance (∼5 nm), suggesting that the domain size is somewhat larger in these directions.

The limited correlation along *c* explains why the background features are so extended in this direction [*cf* Figs. 2 ▸(*e*) and 2(*f*)]. Likewise, the general disorder in the water mol­ecules helps to explain why the trigonal space group appears to fit the pre­sent­ed data. The experiments pre­sent­ed here were performed in TEM mode with a (quasi-)parallel electron beam, hence even with a small condenser aperture, the illumination area still covered approximately tens of nanometres of sample. If the domains of water arrangement are limited to a few nanometres in any direction, then the measurement will sample multiple different water configurations (ordered domains). Averaging across these will likely return the higher sym­metry, as all the possible hy­dro­gen-bonding configurations will be included. This suggests that were the water to arrange perfectly within the field of view, then the diffuse scattering features would no longer be elongated and the increased correlation would make them into stronger superlattice peaks, confirming a potential larger unit-cell size (2*a* × 2*b* × 2*c*) over the original trigonal cell.

## Conclusions

Single-crystal electron diffraction data indicates that the general structure of hydrated rhabdophane can be described using the highest sym­metry trigonal settings, albeit with an effective doubling of the cell com­pared to the original description by Mooney (1948 ▸, 1950 ▸) and considerable displacement of the ion positions within the unit cell that agrees with many of the features of the lower-sym­metry monoclinic structure proposed for this material (Mesbah *et al.*, 2014 ▸). This arises from disorder in the arrangement of the water mol­ecules found in the pores of the structure, that give rise to weak scattering features that could easily be misinter­preted as evidence of more significant sym­metry breaking. Instead, the arrangement of water mol­ecules can initiate randomly at multiple equivalent sites in the structural pore, and when averaged by the relatively large electron beam, this leads to diffuse streaking of the equivalent features in reciprocal space. One future study is to examine the samples at cryogenic tem­per­a­tures to see if removing thermal energy allows the water arrangement to become more regular. Furthermore, there is also likely to be a considerable inelastic scattering arising from the light elements (oxygen and hy­dro­gen) in this structure. The exact difference that this will make to the quality of structure solution is uncertain but with the increasing use of electron-diffraction-based methods, there is a need to better qu­antify these effects and to utilize energy filtering to minimize them in future experiments.

In this study, it was possible to extract the diffuse scattering from within the overall ordered structure to allow PDF analysis of only the disordered sections of the structure to be examined. This allowed the disorder to be attributed solely to positions in the structure describing the network of water occurring in the pores of the mains structure. The ability to perform such single-crystal analysis on nanoscale crystals shows how electron diffraction is an important com­plimentary process to more well-established X-ray and neutron methods. This is particularly relevant when it comes to understanding not only the crystal structure but also the variations within com­plex nanostructured materials.

The results highlight the usefulness of this technique to study order/disorder at nanoscale and therefore its potential in exploring the effects of irradiation-induced structural and phase changes at such scales in the near future.

## Supplementary Material

Crystal structure: contains datablock(s) I, I. DOI: 10.1107/S2053229624007885/eq3020sup1.cif

Unmodified versions of diffraction patterns and schematic diagrams of water ordering. DOI: 10.1107/S2053229624007885/eq3020sup2.pdf

## Figures and Tables

**Table 1:** Recovered lattice parameters from 3D ED of rhabdophane crystals

Parameter	Value
Formula	DyPO_4_·0.667H_2_O
System	trigonal
Space group	*P*3_1_21
*a*	7.019 ± 0.05 Å
*c*	6.417 ± 0.05 Å
Volume	273.78 ± 0.01 Å^3^
Wavelength	0.0251 Å
*R*_2_	0.239
Com­pleteness	91% at 0.7 Å
