# Supplementary material for: 3D electron diffraction studies of synthetic rhabdophane (DyPO4·nH2O)
Source: Acta Crystallogr C Struct Chem. 2024 Sep 4;80(Pt 10):612–9. doi: 10.1107/S2053229624007885 (PMC11451015; doi:10.1107/S2053229624007885)
Supplement: Supplementary file 2 [file c-80-00612-sup2.pdf]

Acta Cryst C STRUCTURAL CHEMISTRY

Volume 80 (2024)

Supporting information for article:

3D electron diffraction studies of synthetic rhabdophane (DyPO4·*n*H2O)

Ercin C. Duran, Mohamed Ruwaid Rafiuddin, Y. Shen, Simon A. Hunt, Anamul Haq Mir and Alexander S. Eggeman

S1. Raw diffraction patterns

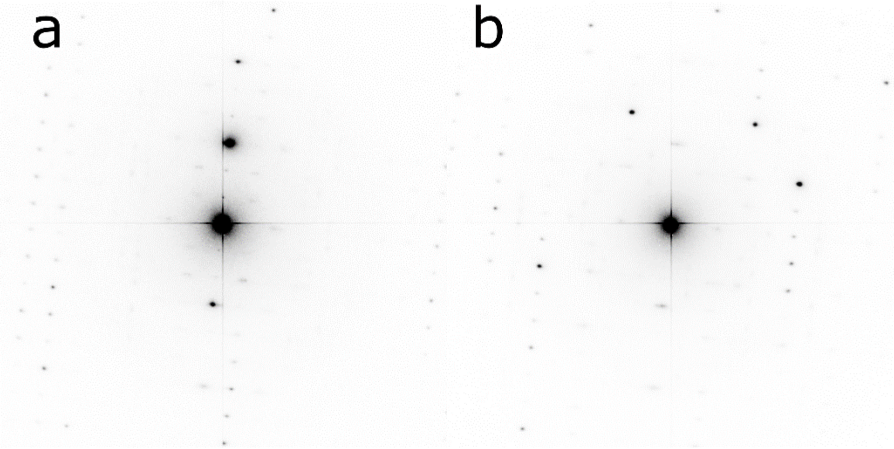

Figure S1. Raw versions of the diffraction patterns shown in Figure 1. Bragg reflections are detectable but background diffuse scattering is virtually non-existent.

For comparison when the natural intensity range of the diffraction patterns are shown, the diffuse background scattering visible between the Laue zones is almost impossible to distinguish.

S2. Water chain disorder models

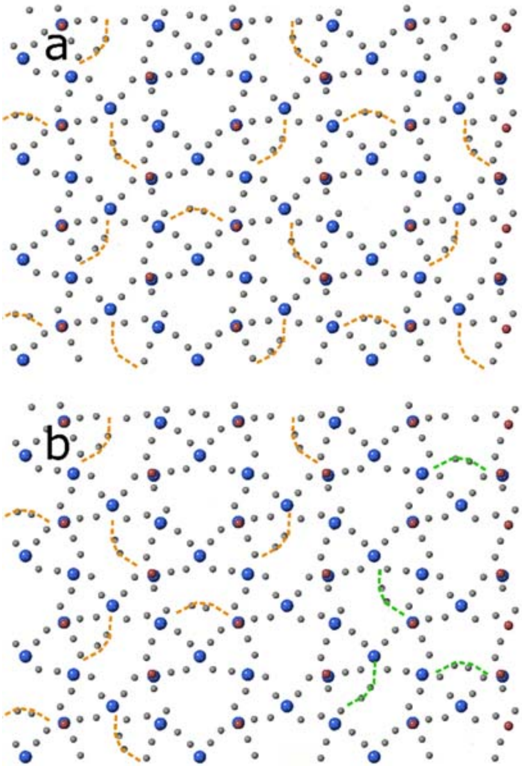

Figure S2. Water order-disorder model. a) shows a possible ordered arrangement of the water chains in the trigonal structure. b) shows a partially correlated arrangement with anti-phase boundaries between regions of different trigonal ordering. The dotted lines indicate the direction of hydrogen bonding between the water molecule chains and the DyO_8 polyhedra in the structure.

In the fully ordered situation in Figure S1a the direction of the hydrogen bonding (indicated with the dotted lines) between the water and the DyO_8 polyhedra in the 1st neighbouring channels are uncorrelated (each domain has an equal chance of putting the water chain in either of the other two possible locations) , however in the second neighbouring channel the hydrogen bonding appears to be fully correlated to the original. Hence this would produce the alternating negative and positive correlation peaks seen in Figures 5b and 5c. The relatively short ordering seen in these experimental PDF suggests that the ordered domains are relatively small (extending over only a few nanometres), a possible disordering is shown in Figure S1b. Here there is a change in the ‘origin’ of the water ordering (the initial hydrogen bonding direction in the channels) leading to an anti-phase boundary in the structure. Changes in the hydrogen bonding direction can also occur along *c*, leading to the limited correlation seen in the pdf maps in this direction.
